# Research and Application Progress of Vegetation Porous Concrete

**DOI:** 10.3390/ma16217039

**Published:** 2023-11-04

**Authors:** Chang Liu, Yangyang Xia, Jianguo Chen, Kai Huang, Jing Wang, Chaojie Wang, Zhuojie Huang, Xunhuai Wang, Cong Rao, Mingsheng Shi

**Affiliations:** 1 Yellow River Laboratory, Underground Engineering Research Institute, School of Water Conservancy and Transportation, Zhengzhou University, Zhengzhou 450001, China; l809664257@163.com (C.L.); 202011221010269@gs.zzu.edu.cn (Y.X.); wang925jing@126.com (J.W.); wangyichaojie123@163.com (C.W.); sms315@126.com (M.S.); 2 Guangxi Key Laboratory of Water Engineering Materials and Structures, Guangxi Institute of Water Resources Research, Nanning 530023, China; gxhuangkai@126.com (K.H.); ph15189803639@163.com (Z.H.); 19851120217@163.com (X.W.); 15108144207@163.com (C.R.); 3 Research Center for Embankment Safety and Disaster Prevention Engineering Technology of Ministry of Water Resources, Yellow River Institute of Hydraulic Research, Zhengzhou 450003, China

**Keywords:** vegetation porous concrete, research progress, material components, mechanical performance, vegetative performance

## Abstract

Vegetation porous concrete is a novel material that integrates concrete technology with plant growth, offering excellent engineering applicability and environmental friendliness. This material is mainly utilized in eco-engineering projects such as riverbank protection, architectural greening, and slope protection along roads. This paper systematically reviews the current research progress of vegetation porous concrete by collecting and analyzing the relevant literature from both domestic and international sources. It covers several aspects including the material components of vegetation porous concrete, such as aggregates, cementitious materials, chemical admixtures, and plant species, as well as aspects like mix design, workability, porosity, pH value, mechanical strength, and vegetative performance. Furthermore, the application of vegetation porous concrete in riverbank protection, slope protection along highways, and urban architecture is discussed, along with a prospective outlook on future research directions for vegetation porous concrete.

## 1. Introduction

Due to traditional concrete materials’ issues such as thermal expansion, thermal contraction, cracking, and lack of permeability, a series of problems such as urban road flooding, land subsidence, and urban heat island effects have become widespread [1]. Consequently, scholars have begun exploring innovative engineering materials to provide better practicality and environmental suitability. In 1852, the United Kingdom developed permeable concrete in urban construction projects [2]. Building on this research, developed countries in the United States and Europe have conducted research and development on green ecological concrete since the late 20th century [3]. Japan started researching concrete materials to improve nutrient-rich water quality in the 1990s. In 1995, the Japan Concrete Institute first proposed the concept of “vegetation porous concrete” and established the Green Concrete Association. Subsequently, they compiled the “Ecological concrete Riverbank Protection Method” [4].

With the acceleration of global urbanization and the increasing prominence of environmental issues, the search for sustainable building materials and engineering solutions has become particularly important. In this context, vegetation porous concrete, as an innovative material that combines plant growth and concrete technology, possesses functions such as water permeability, water purification, dust and noise reduction, soil and water conservation, and environmental beautification [5]. It has gradually attracted more attention. Over the past few decades, vegetation porous concrete has undergone extensive research and application. Many scholars have conducted in-depth research on the performance, mix design, construction process, and other aspects of vegetation porous concrete through laboratory simulations and engineering practices. However, the theoretical framework and application technology of vegetation porous concrete still face many challenges and unresolved issues. Therefore, this paper systematically summarizes the current research progress on vegetation porous concrete by collecting and analyzing the relevant literature from domestic and international sources. It covers several aspects of vegetation porous concrete, including material components such as aggregates, cementitious materials, chemical admixtures, and plant species, as well as mix design, workability, porosity, pH value, mechanical strength, and vegetation performance. Additionally, the paper discusses the applications of vegetation porous concrete in riverbank protection, highway slope protection, and urban construction, and provides a perspective on the future research directions for vegetation porous concrete, aiming to serve as a reference for research and application in the field of vegetation porous concrete.

## 2. The Concept of Vegetation Porous Concrete

**Figure 1:**
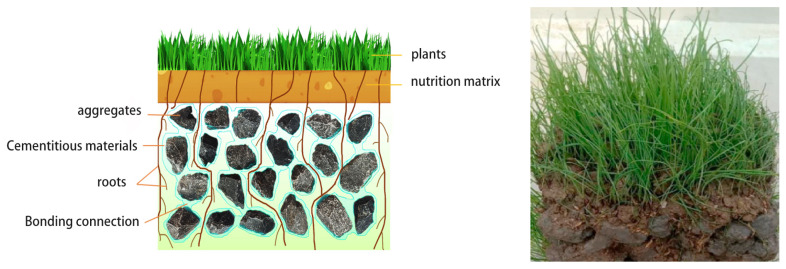
Schematic diagram and physical diagram [10] of vegetation porous concrete.

Vegetation porous concrete (VPC) is a composite material that combines plant growth with concrete technology. It uses concrete as the base material and achieves the integration of plant growth and concrete structure by introducing plants, soil, and other vegetation components into the concrete [6]. Vegetation porous concrete consists of two layers (Figure 1). The bottom layer is a porous concrete composed of coarse aggregates, binding materials (such as cement, concrete, or other mineral materials), and chemical admixtures, while the top layer includes soil, fertilizers, water-retaining agents, and seeds.

The distinctive feature of vegetation porous concrete lies in its dual functionality, combining both plant growth and structural support. The porous structure of vegetation porous concrete provides excellent permeability and breathability [7]. By introducing suitable soil substrate and plant seeds into the concrete, plant roots can grow and establish themselves within the concrete, forming a stable network of plant root systems, thus, achieving the objectives of soil retention, water source protection, and ecological restoration. The mechanical performance of vegetation porous concrete has also been extensively demonstrated in various engineering applications, meeting the requirements for engineering safety and protection [8,9,10]. However, it is worth noting that the construction of vegetation porous concrete demands high technical expertise, has long maintenance periods, and involves limited plant species selection. Therefore, in practical applications, the selection and design of vegetation porous concrete should be made based on specific circumstances, taking into consideration its advantages and disadvantages.

## 3. Composition of Vegetation Porous Concrete

### 3.1. Aggregates

**Figure 2:**
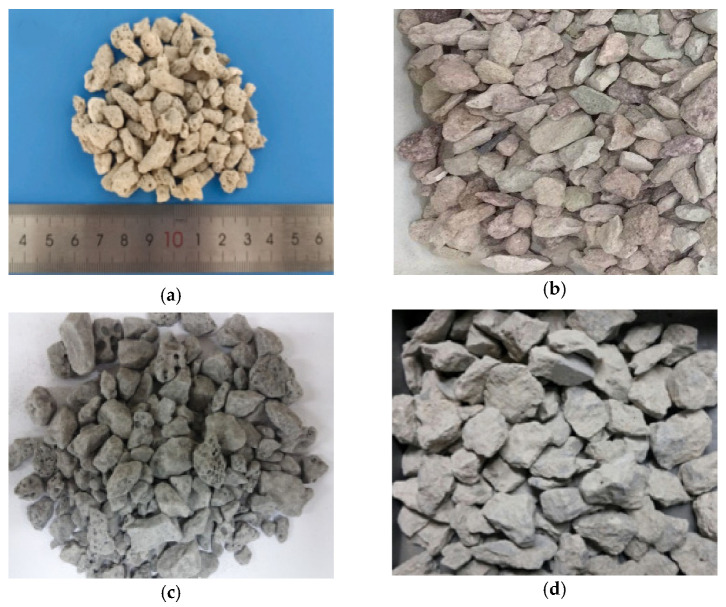
Different types of aggregates: (**a**) coral aggregates [18]; (**b**) zeolite; (**c**) blast furnace slag [7]; (**d**) recycled aggregates [33].

Aggregates constitute the primary component of vegetation porous concrete, occupying the majority of its volume [11,12]. Therefore, the selection of aggregates is crucial for the mechanical performance, permeability, and durability of vegetation porous concrete [13,14]. Currently, various types of aggregates are used in vegetation porous concrete, including high-strength aggregates, lightweight aggregates, purification-type aggregates, and other properties of aggregates [7,15,16,17,18], as shown in Figure 2. In terms of strength, early research on vegetation porous concrete predominantly employed natural aggregates. However, the depletion of natural aggregate resources has prompted researchers to explore alternative resources. For instance, Kim [7] utilized blast furnace slag as an aggregate to prepare vegetation porous concrete, and it was found that the porous nature of the slag promoted vigorous plant growth. In recent years, due to its environmental recycling potential, more attention has been directed towards recycled aggregates. Recycled aggregates are produced by mechanically crushing construction waste, such as discarded concrete and bricks [19]. Recycled aggregates undergo prolonged mechanical wear and chemical degradation, resulting in inferior performance compared to conventional concrete aggregates [20]. Nevertheless, vegetation porous concrete has lower requirements for aggregate strength, and recycled aggregates can meet the strength requirements of vegetation porous concrete [21]. However, numerous studies have indicated that recycled aggregates have a significant impact on the physical properties of vegetation porous concrete [12,22,23,24,25].

Sung conducted a study on vegetation porous concrete made from crushed stone, recycled coarse aggregates, and unsaturated polyester resin. It was found that when the proportion of recycled aggregates mixed with crushed stone was below 50%, the more recycled aggregates were incorporated, the higher the strength of the vegetation porous concrete. This was ultimately confirmed through vegetative testing, showing that planting herbaceous and cool-season grasses on recycled aggregate vegetation porous concrete is feasible [25]. The bonding mortar and interface transition zone in recycled aggregate concrete contain residual old mortar [21], leading to weaker bonding between recycled aggregates and cement. Therefore, the performance of recycled aggregates can be enhanced through special treatment. Tam [19] modified recycled aggregates with acid treatment, and the results show that the water absorption rate of the treated recycled aggregates decreased, while the strength significantly improved. Vegetation porous concrete made with these modified aggregates exhibited significantly increased compressive strength, flexural strength, and elastic modulus.

In the case of lightweight aggregates, He [18] utilized coral waste and seawater to produce multiple sets of permeable concrete with seawater coral aggregates, achieving compressive strength and split tensile strength within the engineering range. Liu [14] used shale granules to prepare vegetation porous concrete and found that as the shale granule content increased, the vegetation porous concrete’s porosity and vegetation performance improved, but the compressive strength decreased.

Concerning water purification, Long [26] conducted experiments using zeolite and ordinary silicate cement to prepare porous vegetation concrete for treating contaminated surface water. Indigenous microorganisms attached to the zeolite aggregate particles were believed to play a significant role in pollutant removal. Yan [13] combined natural zeolite, steel slag, and pumice in pairs to prepare lightweight aggregate vegetation porous concrete for treating eutrophic water. Static tests revealed that the best denitrification and phosphorus removal effect was obtained with a mass ratio of 3:1 for zeolite and steel slag combination.

Apart from aggregate type, aggregate particle size is another parameter that needs to be considered in vegetation porous concrete design. To provide sufficient space for plant growth, coarse aggregates with a particle size ranging from 10 mm to 30 mm are typically used [21,27]. Huang [28] prepared 56 sets of pervious concrete samples with various aggregate types, particle sizes, and mineral admixtures. The experimental results indicate that, within the same aggregate type, smaller aggregate particle sizes typically result in higher strength. Using a single-sized aggregate can increase the porosity of vegetation porous concrete, while a continuous gradation of aggregates is more beneficial for enhancing compressive strength [29,30]. However, coarse aggregates are not the only choice. In reference to Li [31], sea sand was employed as a substitute for river sand, and it was combined with coarse aggregates of specific particle sizes to create sea sand vegetation porous concrete for compressive strength testing. The highest compressive strength achieved was 12.1 MPa, and successful cultivation of tall fescue grass was achieved on it. The aggregate used by various researchers and agencies across the world are summarized in Table 1.

Currently, the selection of aggregates for vegetation porous concrete is moving towards ecological sustainability, with a greater preference for using sustainable and environmentally friendly aggregates to reduce resource consumption, minimize environmental impacts, and achieve better ecological sustainability. The focus in practical engineering applications is on how to turn waste into a valuable resource while considering the source and transportation costs, all while ensuring the performance of the concrete.

### 3.2. Cementitious Materials

Cementitious materials play a crucial role in vegetation porous concrete, serving as the binding and solidifying agents while also providing a conducive environment for plant growth. Different types of cementitious materials possess distinct characteristics that directly impact the performance and functionality of vegetation porous concrete. The most commonly used binding agent, ordinary Portland cement (OPC), is highly alkaline and during hydration, it generates calcium hydroxide phases with a pH value of around 12 [34]. The high alkalinity can potentially harm plant roots, making water absorption difficult, and can also affect the uptake of ions such as calcium, iron, manganese, and boron [35]. Consequently, some research efforts have focused on mitigating the alkalinity issue in vegetation porous concrete by using low-alkalinity cements [36,37].

In the study by Tang [37], low-alkali calcium aluminate cement (CAC) was employed as the primary binding material for vegetated porous concrete. It was observed that the use of CAC resulted in a reduction in the soil pH from 13.5 to 9.9 in the test samples. Furthermore, with the addition of 20% fly ash, the pH could be lowered to 8.7, enabling plants to thrive and grow normally within the vegetated porous concrete. Ming [38] used calcium aluminate cement as the primary binding material to produce concrete specimens. The research found that increasing the gypsum content led to a decrease in alkali content while enhancing the compressive strength of the concrete. The experiments ultimately determined the optimum mix to be 60% calcium aluminate cement, 5% silica fume, 20% gypsum, and 15% mineral powder. Sulfate–aluminate cements have become a recent research focus, and several experimental studies [27,39,40] have indicated that sulfate–aluminate cement-based vegetation porous concrete has a lower pore solution pH value, typically within the range of 10 to 11, as compared to OPC. However, the higher cost of sulfate–aluminate cement has hindered its widespread adoption.

**Figure 3:**
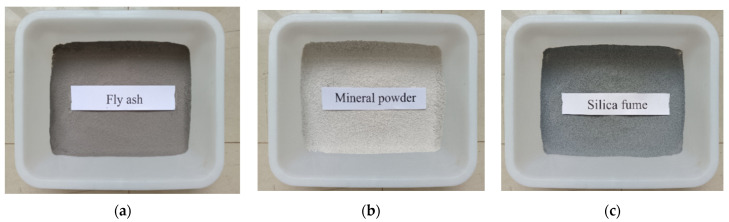
Different types of mineral admixtures: (**a**) fly ash; (**b**) mineral powder; (**c**) silica fume.

Furthermore, the use of mineral admixtures such as fly ash [37,41,42], mineral powder [38,43,44], and silica fume [38,45], as shown in Figure 3, or waste like steel slag [46] and charcoal powder [47] as supplementary cementitious materials (SCMs) in lieu of traditional cement not only reduces costs but also benefits the environment [20]. Ganapathy [42] investigated the influence of fly ash and silica fume on the planting characteristics of vegetation porous concrete, confirming that both fly ash and silica fume could enhance plant growth by improving alkalinity. In the research conducted by Zhao [47], different mass ratios of biochar were incorporated into vegetated porous concrete, and the trends in porosity, permeability, and plant adaptability were studied. The study reveals that the addition of an appropriate amount of biochar improves the soil environment and promoted plant growth. However, it also leads to the closure of pores in vegetated concrete, resulting in adverse effects such as reduced porosity and permeability. The experiments concluded that the optimal biochar content was 5 kg/m^3^. The findings from different research studies are summarized in Table 2.

In summary, researchers are actively seeking and developing alternative binding materials to replace ordinary Portland cement (OPC) in order to enhance the performance and environmental sustainability of pervious concrete. These efforts aim to provide more eco-friendly and sustainable options for the application of pervious concrete, thus, contributing to the reduction in adverse environmental impacts in construction and infrastructure projects.

### 3.3. Chemical Admixtures

Chemical admixtures come in a variety of types, each with its own functions and characteristics. Due to the specific requirements of vegetation porous concrete in terms of mechanical strength, workability, and environmental pH levels, the use of admixtures can enhance concrete performance, improve its engineering properties, and enable better performance in various application areas. High-efficiency water reducers [9,10,11,12] are used to enhance the fluidity of cement slurries, making it easier for the cement slurry to encapsulate the aggregates. Yang [48] soaked and sprayed vegetation porous concrete with oxalic acid, which effectively lowered the pH of the vegetation porous concrete. However, compared to spraying, soaking significantly reduced the mechanical strength of the vegetation porous concrete. Bao [49] developed a self-made additive for vegetation porous concrete, mainly composed of chemical components such as silica fume and high-efficiency water reducing agent. It not only effectively reduces the alkalinity of cement, but also increases the strength of cement to 25.2 MPa at a dosage of 3.6%.

Currently, polymers are added as an admixture to vegetation porous concrete due to their excellent alkali-reduction effects. Kim [8] added butadiene latex as an admixture and found that it improved both porosity and compressive strength of the vegetation porous concrete. However, its freeze–thaw resistance decreased compared to concrete without latex. Didier Snoeck [50] introduced superabsorbent polymers (SAP) to improve the water retention and implantation characteristics of the cementitious materials, observing that SAP enhanced the biological acceptance and had broad applicability. Chen [16] investigated the preparation of recycled aggregate vegetation porous concrete by partially substituting cement with volume fractions of various polymers such as butadiene latex, styrene butadiene latex, and waterborne polyurethane. Styrene butadiene latex, with a 5% addition, exhibited outstanding compressive mechanical performance and effectively reduced the pH of the pore solution in the concrete, demonstrating excellent vegetative properties. Li [51] used SAP to modify natural soil and found that with increasing SAP content, soil erosion resistance significantly improved, and soil water absorption and retention properties were noticeably enhanced. However, the use of SAP did not substantially affect the soil’s alkalinity, and high SAP dosage levels inhibited plant germination. The characteristics of chemical admixtures modified VPC are summarized in Table 3.

With the continuous development of vegetation porous concrete technology, a range of specialized admixtures designed specifically for vegetation porous concrete [52,53,54] have been developed. An increasing number of chemical admixtures are being explored for addition to vegetation porous concrete, with functionalities extending beyond enhancing mechanical strength and reducing alkalinity. These additives aim to promote vegetation, facilitate construction, and introduce additional properties into the mix.

### 3.4. Selection of Plants

**Figure 4:**
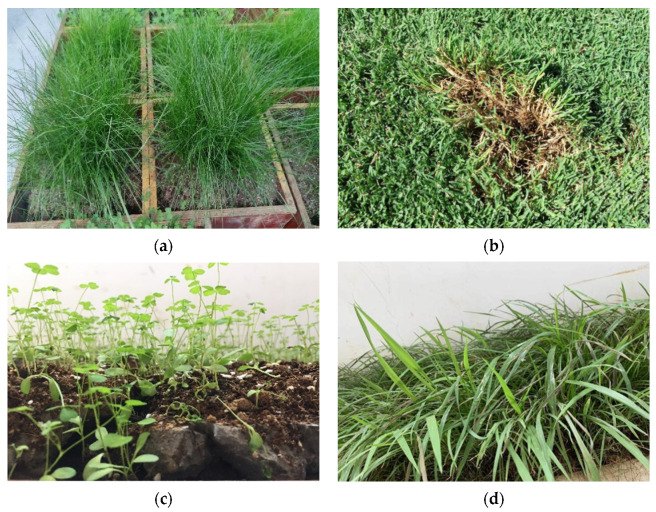
Different plant varieties: (**a**) tall fescue; (**b**) Bermuda grass [59]; (**c**) alfalfa; (**d**) *Paspalum wettsteinii*.

Plants play a crucial role in maintaining ecological balance, protecting soil and slopes. However, due to variations in the growth characteristics of different plant species, the selection of plant species is paramount for their survival and growth in vegetation porous concrete. Currently, commonly used test plants such as tall fescue [31,40,43], Bermuda grass [40,43], and purple alfalfa [31,40,43,55] are often chosen for vegetation porous concrete research due to their alkali resistance, ease of cultivation, and robust root systems. The above-mentioned plant varieties are shown in the Figure 4a–c.

Some researchers have proposed that the best approach is to diversify species, such as combining warm-season and cool-season grasses, as this can enhance the greening effect [55,56]. Chen [57] planted a combination of tall fescue, Bermuda grass, Puccinellia, and three other mixed grass species in porous concrete blocks and found that the mixed grass combination named LanLanJing III yielded the best greening effect. Fan [58] used a mixed seed combination of warm-season Bermuda grass and cool-season tall fescue to restore vegetation on existing concrete slopes, achieving significant results with plants sprouting and growing alternately throughout the year.

Local species not only better adapt to local climates and soils but also prevent species invasions. Tang [37], in the absence of prior vegetation porous concrete research in Australia, conducted studies using three native Australian plants: *Elymus scaber*, *Themeda trianda*, and *Chloris truncata*. They found that kangaroo grass and windmill grass were suitable for vegetation porous concrete technology, while clover was relatively incompatible. Chen [16] selected local plant *Paspalum wettsteinii* in Guangxi, as shown in Figure 4d, and planted them together with traditional research plants such as Bermuda grass and tall fescue. The results show that *Paspalum wettsteinii* grew better in planting concrete compared to other plants. The results obtained from different research studies are summarized in Table 4.

Currently, most vegetation porous concrete research predominantly focuses on herbaceous plants. This preference is attributed to the adaptability, robust root systems, extensive distribution, wear resistance, and aesthetic qualities commonly found in herbaceous plants, making them ideal for vegetation porous concrete applications in slope protection and road surfaces.

## 4. Experimental Investigation of Vegetation Porous Concrete

### 4.1. Mix Design

Unlike traditional concrete, the mix design of vegetation porous concrete presents a contradiction between the pH environment required for plant growth and the high alkalinity of concrete. It also involves the simultaneous need for high porosity and sufficient mechanical performance [51]. The mix proportion of vegetation porous concrete needs to consider factors such as the selection and proportion of coarse and fine aggregates, the type and amount of cementitious materials, and the type of plants and their growing environment. The goal is to achieve the target porosity and pore size distribution that can support plant growth and root development [60,61]. The water–cement ratio and paste–aggregate ratio are significant parameters, with the former ranging between 0.25 and 0.35, and the latter ideally adjusted between 0.13 and 0.20. In the process of mix design, the choice of mix design calculation method can significantly impact the final design parameters. One commonly used mix design calculation method is the absolute volume method, which is based on the volumetric proportions of each component in concrete. This method allows for the control of the distribution and size of pores within the concrete [62]. However, when using the volume method for the mix design of vegetated ecological concrete, discrepancies between the measured porosity and the target porosity can arise as the slurry volume increases [45].

In addition to the volume method, mix design calculation methods for vegetated concrete include the mass method and the specific surface area method. The mass method is based on the mass proportions of the various components in concrete and offers the advantages of speed and efficiency in calculations. However, it may not be as effective in controlling slurry volume. The specific surface area method [63] calculates the amount of binder required for a unit volume of porous concrete based on the surface area within a unit volume of aggregate. This method is aligned with the theoretical concept of binder enveloping aggregate, but the mix design process can be more complex and intricate.

While vegetation porous concrete has been extensively studied, mix design still faces challenges, including the use of empirical coefficients and overly idealistic assumptions. To address these issues, Wang [43] attempted a different approach by first creating cement mortar specimens for compression and flexural tests. This approach determined the optimal amount of ultrafine slag, facilitating subsequent mix design experiments. Additionally, Wang G [63] improved the concept of binder-to-aggregate ratio, and Li combined the volume method with the mass method in mix calculations [18]. These attempts have shown promising improvements in mix design.

### 4.2. Fluidity

Slurry flowability is an essential indicator for assessing the workability of vegetation porous concrete mixtures. Excessive flowability in the cementitious slurry can lead to the phenomenon of slurry pooling at the bottom of the specimen during the initial molding stage. Conversely, if the flowability is too low, the cementitious slurry may not evenly envelop the surface of coarse aggregates, affecting the concrete’s pore structure and ultimately reducing its compressive strength. Therefore, the flowability of the paste in vegetation porous concrete is crucial for ensuring the uniformity, workability, and fillability of the material during construction. Several factors influence the flowability of the paste in vegetation porous concrete, including the water–cement ratio [32,64], additives [65], cement material quality [66], equipment, temperature, and environmental conditions [67]. The combined effect of these factors determines the flow performance of the paste.

Due to the relatively recent initiation of research on vegetation porous concrete in several regions, there is a lack of unified and universally recognized specifications and methods [21]. Additionally, regulations governing the flowability of vegetation porous concrete are generally absent. In situations where variations in raw materials exist, researchers are compelled to perform multiple experiments to ascertain the suitable range of flowability. This presents substantial challenges to both experimental research and engineering applications.

Regarding the recommended range for the flowability of the paste in vegetation porous concrete, Wang [63] suggests that it should be between 160 mm and 200 mm to ensure sufficient bonding between the aggregate contact points without excessive sedimentation of the cementitious paste. Wu [68] conducted a systematic study on the flowability of cement paste in vegetation porous concrete and found, based on the flowability test conducted in accordance with GB/T 2419-2016 [69], that when the flowability of the paste is between 210 mm and 240 mm, the porosity and permeability coefficient remain relatively stable, and the compressive strength reaches the target design strength. Gong [70], referring to GB/T 8077-2000 [71], tested the flowability of sulfate–aluminate cement slurry and used a flowability of 80–160 mm to prepare vegetation porous concrete. The compressive strength of vegetation porous concrete is highest when the flowability is between 60 mm and 80 mm.

Currently, different testing methods and conditions have a significant impact on the results of measuring the flowability of the cementitious paste in vegetation porous concrete.

### 4.3. Porosity

**Figure 5:**
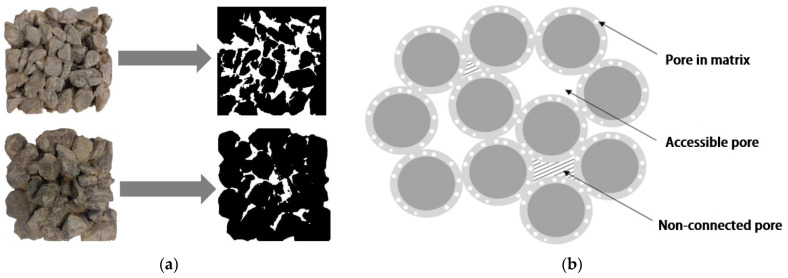
(**a**) Image processing of vegetation porous concrete section [32]; (**b**) connected pores and ineffective pores [75].

Porosity is a key indicator for assessing the performance of vegetation porous concrete [20], influenced by factors such as aggregate particle size [14], paste–aggregate ratio [72,73], and type of cementitious materials [27]. Porosity not only affects the mechanical strength of vegetation porous concrete but also plays a crucial role in plant growth, directly impacting water supply, gas exchange, and root development. The Japanese method for eco-concrete revetments specifies that porosity should be controlled within the range of 21% to 30%, and porosity is inversely proportional to the mechanical strength of vegetation porous concrete [72]. This has been confirmed by experiments conducted by Wang [43], in which increasing porosity from 15% to 35% resulted in a decrease in compressive strength from 24 Mpa to 4 Mpa and a decrease in split tensile strength from 2.2 Mpa to 0.8 Mpa. Porosity also affects the distribution of pore space [14]. Wang [43] used industrial CT scans for non-destructive testing of vegetation porous concrete and measured pore parameters using VG software. The experiments concluded that a higher porosity leads to a larger pore volume and a higher frequency of large pores. Additionally, larger aggregate particle sizes result in tighter pore connectivity and a sparser structure. Chen [32], in order to analyze the surface porosity of vegetation concrete, employed a scalpel to section cubic standard specimens into layers. Digital photographs of the concrete sections were captured, and subsequent image processing involved equalization and grayscale conversion, as shown in Figure 5a. The experiment found that under the same water–cement ratio, the larger the particle size of the aggregate, the greater the porosity.

Vegetation porous concrete contains discontinuous pores, which hinder the flow of water and nutrients. Sung-Bum Park [74] proposed the use of effective porosity and total porosity to differentiate between voids in pervious concrete. Total porosity refers to the proportion of all pore space in the concrete, including connected and disconnected pores. Effective porosity refers specifically to the proportion of connected pores that have direct contact with water, gas, and other media, reflecting the permeability and transport properties of liquids and gases in the concrete. Effective porosity is smaller than or equal to total porosity in porous concrete, and considering effective porosity allows for a more accurate prediction of compressive strength in porous concrete [75], the relationship between effective pores is shown in Figure 5b.

Ultimately, porosity is formed by the irregular arrangement of aggregates and serves the purpose of permeability and vegetative performance. With the mechanical strength meeting the requirements of the engineering design, a larger aggregate particle size results in a higher porosity [64], providing more space for plants, soil, and water. Effective porosity, on the other hand, relates to fluidity, and naturally, a larger effective porosity is preferable.

### 4.4. pH

Reducing the pH of vegetation porous concrete is primarily aimed at providing a suitable soil environment for plant growth. Most plants thrive within a pH range of 3.5 to 8.5 [20]. Currently available techniques for reducing pH have their limitations, including:

1. Using acidic or neutral supplementary cementitious materials in place of cement to lower cement content and potentially recycle waste materials. However, the application of supplementary cementitious materials to reduce the alkalinity of vegetation porous concrete is limited [76,77];
2. Utilizing different types of low-alkalinity cements, which can be more costly compared to ordinary Portland cement [8];
3. Immersing vegetation porous concrete in acidic solutions such as ferrous sulfate, oxalic acid, and ammonium phosphate to neutralize alkaline components within the concrete. While this approach can reduce alkalinity, it has a significant impact on the mechanical and durability properties of the concrete [78], and its application during construction can be challenging;
4. Selecting the addition of chemical admixtures, which has proven to be efficient [8,48,49,50,51,52,53,54]. However, environmental impacts, durability, higher costs, research, regulation, and monitoring are important considerations and challenges associated with this method.

Determining pH is a crucial step in assessing the acidity or alkalinity of vegetation porous concrete and the soil environment. Accurate pH measurements provide valuable insights for adjusting plant growth and concrete conditions. Common pH testing methods include alkalinity precipitation and solid–liquid extraction [34]. Both methods involve measuring the pH of the soaking water solution of vegetation porous concrete samples.

In the alkalinity precipitation method [58], 6.0 kg of distilled water is added to a 20 cm container, and the mixture is left to stand for 24 h.

The solid–liquid extraction method [14] involves grinding and sieving the sample, adding ten times the mass of distilled water, and shaking it every 5 min. The pH of the water solution is then measured after 2 h.

### 4.5. The Mechanical Strength Characteristics of Vegetation Porous Concrete

The mechanical strength characteristics of vegetation porous concrete can be analyzed and described from three perspectives: macro level, meso level, and micro level. The comprehensive assessment from these perspectives determines the overall mechanical performance and durability of vegetation porous concrete [79]. From a macroscopic point of view, vegetation porous concrete exhibits certain mechanical properties such as compressive strength, tensile strength, and flexural strength. These properties can be evaluated through conventional mechanical tests, such as compression tests, tensile tests, and flexural tests. Scholars generally attribute the failure of vegetation porous concrete to insufficient cementitious bonding properties [16]. Unlike the brittle failure mode of conventional concrete, vegetation porous concrete demonstrates a degree of ductility during the failure process. Under mechanical loading, cracks first appear at the four corners of the stressed surface, and then these cracks propagate and expand vertically along the vertical edges, gradually forming vertical primary cracks. These vertical primary cracks extend outward along the vertical edges until failure occurs.

**Figure 6:**
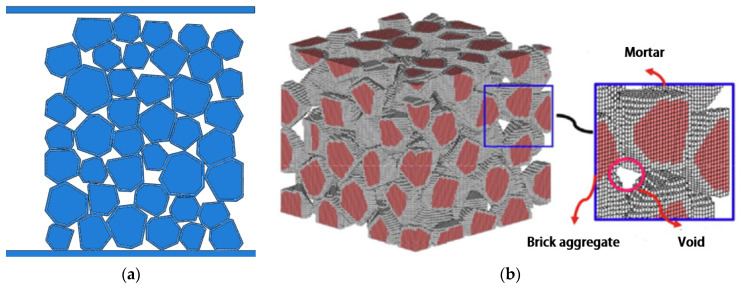
(**a**) A two-dimensional model of vegetation porous concrete; (**b**) three-dimensional numerical model of vegetation porous concrete [11].

From meso level, vegetation porous concrete is a composite material composed of cementitious materials and aggregates. Microscopic structural features include the distribution of aggregates, the interface bonding between aggregates and the cementitious matrix, and the distribution of pores, all of which affect the strength and durability of vegetation porous concrete. In recent years, with the continuous development of computer technology and numerical simulation methods, finite element analysis software has been applied to the field of mechanical analysis. The correctness of model establishment has a significant impact on the final results. Xu [80] made a contact algorithm for aggregates and produced a two-dimensional model of vegetation porous concrete. The two dimensional model of vegetation population concrete is similar to Figure 6a. Wang [43] used CT scanning to reconstruct a three-dimensional model of vegetation porous concrete. Ma [11] used an aggregate placement algorithm to construct a three-dimensional microscopic model that considered the random shape and size of aggregates. The commercial software ANSYS version 17.2 and LS-DYNA. was used to analyze the influence of porosity on the internal stress of porous concrete. The results indicate that the collapse of aggregates caused by the fracture of cementitious materials is the main failure mode of vegetation porous concrete under uniaxial compression. By comparing numerical simulations with experimental results, the reliability of the three-dimensional simulation method and model parameters in studying the compressive properties of vegetation porous concrete was verified, as shown in Figure 6b.

**Figure 7:**
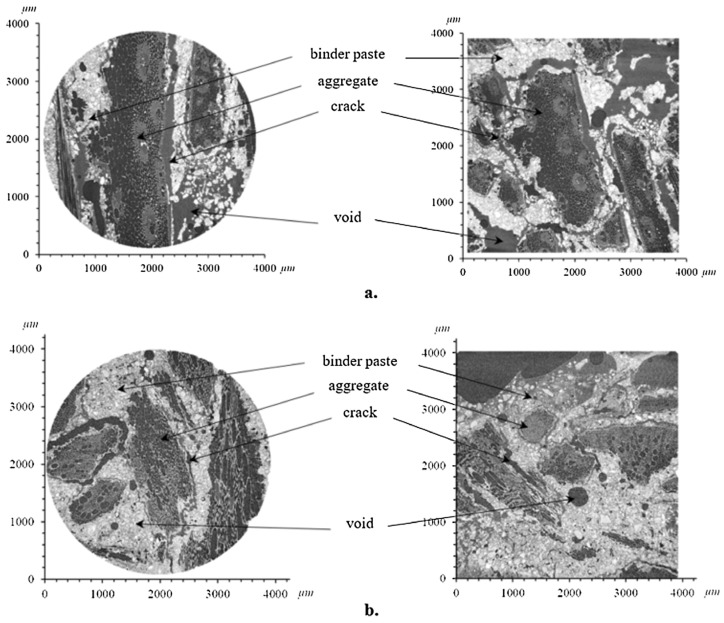
X-ray tomography images [82]: (**a**) corn biological substrate; (**b**) sunflower metakaolin (voxel size 4 mm).

From micro level, the mechanical strength characteristics of vegetation porous concrete are influenced by the interactions among its various components within the microstructure. The hardening process of cement involves hydration reactions and crystal formation, leading to an increase in the strength of the cementitious material [81]. Additionally, other added materials may either promote or interfere with the hydration of cement. Méryl Lagouin [82] used corn and sunflower residues as biobased materials, and lime with kaolin as binders to prepare bio-based aggregate-based concrete. To better understand the stress–strain behavior of the material, X-ray tomography scans were conducted on the kaolin-based concrete, as shown in Figure 7. The test results indicate the presence of shrinkage cracks at the interface between the bio-based aggregate and the binder paste. This implies that even in the absence of external loading, the concrete exposed to a dry environment experiences shrinkage, leading to increased tensile stress and subsequent cracking. In the absence of external loading, concrete exposed to dry environments undergoes shrinkage, leading to increased tensile stresses and crack formation. Shen [46] used scanning electron microscopy (SEM) to observe steel slag vegetation porous concrete specimens with phosphogypsum as a variable. In SEM images of the carbonated consolidated matrix containing 2.5% phosphogypsum, symmetric hydration products (calcium aluminate, hydrated gel) were observed. Carbonation resulted in the generation of a significant amount of calcium carbonate, with more interconnected and denser crystals, indicating a positive effect of reasonable phosphogypsum incorporation on the carbonation of steel slag.

The mechanical strength characteristics of vegetation porous concrete directly impact its performance and durability in various engineering applications. By understanding and optimizing these properties, it is possible to design vegetation porous concrete structures that are stronger, more stable, meet engineering requirements, and enhance their service life.

### 4.6. The Influence of Plants on Vegetation Porous Concrete

Vegetation porous concrete and plants have a mutually influential and constraining relationship. The presence of plants can lead to changes in various properties of vegetation porous concrete. Stresses generated by the expansion of plant roots have the potential to disrupt the physical structure of vegetation porous concrete, and the moisture introduced by plants can cause erosion of the concrete [56], resulting in a reduction in mechanical strength [15].

**Figure 8:**
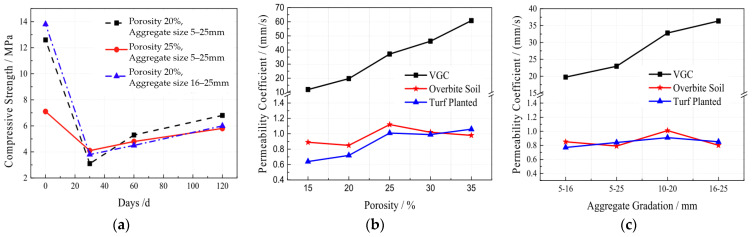
Changes in vegetation porous concrete after planting plants: (**a**) compressive strength [43]; (**b**) permeability coefficient [43]; (**c**) frost thawing resistance [43].

Wang [43] conducted a comprehensive study on the impact of plants on vegetation porous concrete. It was observed that during the initial 28 days after planting, the compressive strength of vegetation porous concrete significantly decreased due to plant growth, followed by a gradual recovery. Increasing the porosity to a certain extent reduced the weakening effect of plants on strength, as shown in Figure 8a. After planting, the permeability of vegetation porous concrete was similar to that after soil cover, with a significant decrease in the permeability coefficient, although it remained higher than that of regular soil, as shown in Figure 8b. Several days after planting, the compressive strength of vegetation porous concrete did not show a significant decline compared to before freeze–thaw cycling, indicating that planting vegetation can enhance the frost resistance of vegetation porous concrete, as shown in Figure 8c.

Currently, there is limited research on the impact of plants on vegetation porous concrete. When designing vegetation porous concrete, it is essential to understand plant characteristics and consider the potential negative effects of plants on concrete strength to ensure the performance and sustainability of vegetation porous concrete.

## 5. Application of Vegetation Porous Concrete

**Figure 9:**
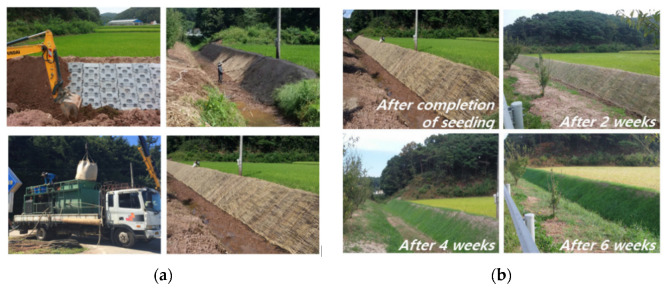
Slope application of vegetation porous concrete: (**a**) repair process [83]; (**b**) construction application effect [83].

The combination of vegetation and concrete not only balances strength and aesthetics but also promotes ecological cycling, making it an essential choice in today’s sustainable development trends. Vegetation porous concrete effectively prevents soil erosion and slope instability, providing natural vegetation cover, which plays a significant role in river ecosystem restoration and water purification. It has been widely applied in riverbank engineering and roadside slope protection. Kim [83] used vegetation porous concrete blocks on the slopes of a riverbank, covered them with a 10 cm layer of soil, and sowed various plant seeds. Monitoring the plots for 6 weeks showed vigorous plant growth. Figure 9 illustrates the process of project repair and the application’s effectiveness.

**Figure 10:**
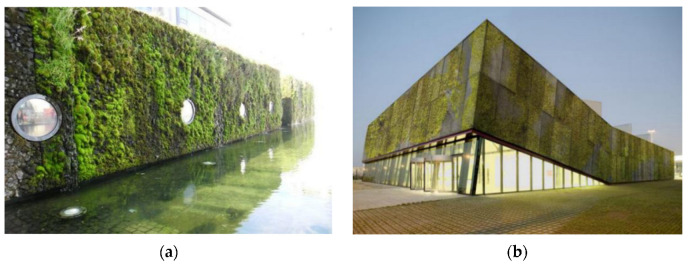
(**a**) The moss wall of the Reykjavik City Hall [9]; (**b**) concept of vegetation porous concrete office building construction [9].

In the field of architecture, vegetation porous concrete offers designers greater creative freedom. It not only fosters innovation in architectural aesthetics but also promotes the integration of buildings with the natural environment, as depicted in Figure 10a. Experimental research has confirmed the suitability of lightweight aggregate porous concrete [84] for rooftop applications. Li [85] utilized the porous concrete vegetation bricks developed by his team in vertical green walls and rooftops.

The Polytechnic University of Catalonia [9] has pioneered a low-pH gravel substrate for the cultivation of blue–green algae, green algae, and mosses. They have designed specialized multilayer systems capable of collecting rainwater and storing it within the material’s microscopic structure to prevent moisture loss. This material can serve as decorative elements in architecture, as illustrated in Figure 10b. The dream of lush green gardens has long been part of human aspiration, and vegetation porous concrete presents a practical solution for incorporating living nature into urban architecture.

## 6. Conclusions and Future Prospective

Vegetation porous concrete possesses excellent permeability, suitable vegetative capabilities, and tremendous environmental potential. Its application in riverbanks and roadside slopes has matured, while in the construction industry, it provides a visually impactful green material with vast prospects. However, as vegetation porous concrete is a relatively new technology that has flourished in the past two decades, it faces persistent barriers to research and development:

1. A lack of unified standards and guidelines for vegetation porous concrete hinders its research and application. It is imperative to promote the development and publication of national or regional standards for vegetation porous concrete.

There are several gaps in the current research on vegetation porous concrete;

2. Presently, vegetation porous concrete predominantly favors herbaceous plants, which significantly affect material strength due to their pore space requirements. Algae and mosses, in contrast, require less pore space for growth, offering a new direction to address the trade-off between strength and porosity;
3. Soil serves as the foundation for plant growth, yet current research primarily focuses on mix design and alkali reduction in vegetation porous concrete, with limited study on the soil used to fill gaps. Selecting suitable plant–soil combinations and conducting improvements may be key to successful research in vegetation porous concrete.
4. Most research on vegetation porous concrete concentrates on conditions before plant growth. However, post-planting factors, such as the forces exerted by plant roots, substances secreted by plants, and soil microorganisms, require consideration and study.

Looking ahead, there are promising prospects for the less explored applications of vegetation porous concrete:

5. The use of vegetation porous concrete for road surface paving in projects that prioritize ecological conservation and environmental beautification holds wide-ranging potential. Despite its potentially lower strength compared to regular concrete road surfaces, it offers advantages such as permeability, heat island effect mitigation, and air purification;
6. Vegetation porous concrete is predominantly applied horizontally, with limited attention given to vertical structures. The judicious utilization of spatial resources may represent an opportunity for the development of vegetation porous concrete.

## Figures and Tables

**Table 1:** Summary of aggregates used in vegetation porous concrete.

Source	Aggregate Type	Aggregate Size	Aggregate Density /kg·m^3^	Porosity/%	28 d–Compressive Strength (Mpa)
Chen 2022 [32]	Limestone	10–40	2690–2710	41.8–44.3	5.2–8.1
Ma 2021 [11]	Brick aggregate	20–40	1740–1800	44–46	0.71–1.73
He 2023 [18]	Coral aggregate	2.36–9.5	2036	48.7	6.2–18.5
Wang 2023 [22]	Recycled aggregate	5–25	2390–2410	44.2	2–24
Kim 2016 [7]	Blast furnace slag	8–25	2360	—	9.5–13.5

**Table 2:** Summary of cementitious materials used in VPC.

Source	Type of Cementitious Materials	Type of SCMs	pH	Optimal Ratio
Tang 2018 [37]	CAC	Fly ash (FA)	8.7	20%FA
Li 2023 [27]	SAC	Bentonite	10.93	SAC with 2% bentonite
Chen 2023 [45]	Red mud	Silica fume (SF), blast furnace slag (BFS), and diatomite (DA)	Less than 9	10% SF and 5% DA
Wang 2019 [43]	P.O 42.5 ordinary Portland cement	Ultrafine slag (UA)	10 and 8 (acid treatment)	40% UA

**Table 3:** Characteristics of chemical admixtures modified VPC.

Source	Chemical Admixtures	Function	Findings
Zhang 2022 [10]	Ethylene–vinyl acetate copolymer (EVA)	Increase the cohesive capacity between aggregates.	The good resistance of concrete to dry and wet cycles is due to the addition of EVA to form a polymer film. EVA has outstanding waterproof properties, which can prevent material degradation and effectively bond cement hydrates and aggregates
Yang 2016 [48]	Oxalic acid	Reduce alkalinity	When it comes to reducing pH using oxalic acid, immersion is more effective than spraying. However, immersion results in a decrease in the compressive strength of the concrete.
Yang 2016 [48]	Silica fume and ferrous sulfate	Reduce alkalinity	Compared to single alkali reduction treatment, the combined alkali reduction treatment significantly influences the strength of the specimens, indicating that alkali reduction treatments carried out during the preparation process have a greater impact on the strength of porous concrete.
Didier Snoeck 2022 [50]	Superabsorbent polymers (SAP)	Improving water retention and bio-receptivity	Due to the water retention capacity of the SAPs creating an ideal situation for algal colonization
Chen 2022 [16]	Butadiene latex, styrene butadiene latex, and waterborne polyurethane	Reduce alkalinity	Styrene butadiene rubber latex and styrene acrylic lotion can effectively reduce the pH value of concrete pore solution, and the greater the amount of styrene butadiene rubber latex and styrene acrylic lotion, the more obvious the effect of alkali reduction is. On the contrary, waterborne polyurethane lotion will have the opposite effect.

**Table 4:** Summary of plants planted on VPC.

Source	Plant Species	Planting Area	Major Conclusions
Yan 2016 [13]	Bahia grass	Guangdong, China (20–25° N)	A 1:1 to 1:3 mixture of zeolite and pumice in the vetiver grass vegetation porous concrete exhibits excellent denitrification and phosphorus removal effects.
Tang 2018 [37]	*Elymus scaber*, *Themeda trianda*, and *Chloris truncata*	Australia	*Chloris truncata* demonstrates a significantly better adaptability to the concrete environment compared to the other two tested grass species.
Chen 2022 [15]	Bermuda grass, Puccinellia, alfalfa, *Paspalum wettsteinii*	Guangxi, China (20–26° N)	In comparison to Bermuda grass, Puccinellia and alfalfa, *Paspalum wettsteinii* is better adapted to the climate characteristics of Guangxi and the unique survival environment of vegetation porous concrete
Sung 2010 [25]	Tall fescue, perennial ryegrass, Lespedeza	South Korea (34–38° N)	Regardless of the vegetation type, the initial germination time is almost similar, ranging from 4 to 7 days, and subsequent growth is good

## Data Availability

Data will be made available on request.
